# Antimicrobial activity of IDD-B40 against drug-resistant *Mycobacterium tuberculosis*

**DOI:** 10.1038/s41598-020-80227-y

**Published:** 2021-01-12

**Authors:** Md Imtiazul Islam, Hoonhee Seo, Sukyung Kim, Venkata S. Sadu, Kee-In Lee, Ho-Yeon Song

**Affiliations:** 1 Department of Microbiology and Immunology, School of Medicine, Soonchunhyang University, Cheonan, 31151 Chungnam Korea; 2 Probiotics Microbiome Convergence Center, Soonchunhyang University, Asan, 31538 Chungnam Korea; 3 Green Chemistry Division, Korea Research Institute of Chemical Technology, Daejeon, 34114 South Korea; 4 Major of Green Chemistry and Environmental Biotechnology, University of Science and Technology, Daejeon, 34113 South Korea

**Keywords:** Drug discovery, Microbiology

## Abstract

The emergence of multi-drug resistant (MDR) and extensively drug-resistant (XDR) *Mycobacterium tuberculosis* creates the urgency for new anti-tuberculosis drugs to improve the efficiency of current tuberculosis treatment. In the search for a new potential tuberculosis drug, we synthesized an isoindole based chemical library and screened a potential candidate with significant anti-tuberculosis activity. The compound named 2-hydroxy-4-(4-nitro-1,3-dioxoisoindolin-2-yl) benzoic acid (IDD-B40) showed strong activity against all the tested drug-susceptible and drug-resistant strains of *M. tuberculosis,* with the 50% minimum inhibitory concentrations (MIC_50_) of 0.39 μg/ml both in culture broth and inside Raw 264.7 cells. Also, IDD-B40, in combination with rifampicin, exhibited a direct synergistic effect against both XDR and H37Rv *M. tuberculosis*. Besides, IDD-B40 showed a better post-antibiotic effect (PAE) than did some first-line drugs and showed no significant cytotoxicity to any cell line tested, with a selectivity index of ≥ 128. Although IDD-B40 showed a result similar to isoniazid in the preliminary mycolic acid inhibition assay, it did not exhibit any effect against other mycolic acid-producing nontuberculous mycobacterial strains (NTM), and different non-mycobacterial pathogenic strains, so further studies are required to confirm the mode of action of IDD-B40. Considering its results against *M. tuberculosis*, IDD-B40 is a potential anti-tuberculosis drug candidate. However, further studies are required to evaluate its potential in vivo effect and therapeutic potential.

## Introduction

Tuberculosis is one of the oldest and most highly contagious diseases caused by *Mycobacterium tuberculosis* (*M. tuberculosis*). It is still one of the leading causes of global death, claiming 1.5 million lives, with around 10 million new cases in 2018^1^. Bedaquiline, linezolid, and pretomanid are the only drugs that have been approved recently, after nearly five decades of the first-line TB chemotherapy. However, first-line drugs are still the primary treatment of choice for drug-susceptible *M. tuberculosis* infection^2,3^. Nevertheless, these drugs are facing significant compliance challenges because of the long duration of therapy and associated toxicities^4^. Besides, the emergence of multidrug-resistant (MDR) and extensively drug-resistant (XDR) *M. tuberculosis* has taken this global concern to a new height with 484,000 diagnosed drug-resistant tuberculosis infections in 2018^1,4^. Thus, new drug candidates that can shorten current first-line drug regimens and provide effective therapies against both drug-sensitive and drug-resistant TB are urgently needed^5^.

A series of indole-based compounds possessing anti-tuberculosis activity was reported during the last decade^6–8^. To extend the research on indole as a new class of anti-tubercular agent, we designed a chemical library based on indole. A total of 25 indole derivatives were synthesized at Korea Chemical Bank of Korea Research Institute of Chemical Technology, Korea and initially tested for in vitro anti-tubercular activity against *M. tuberculosis* H37Rv and H37Ra. After an extensive screening test, we found a promising isoindoledione based compound, 2-Hydroxy-4-(4-nitro-1,3-dioxoisoindolin-2-yl) benzoic acid (IDD-B40), that has an intense activity against *M. tuberculosis*. Overall, we selected it for further studies to find out whether it might have characteristics suitable as a potential anti-tuberculosis drug.

## Materials and methods

### Synthesis of IDD-B40

**Figure 1:**
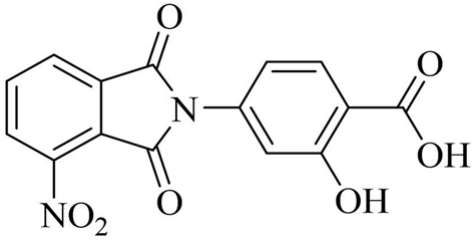
Chemical structures of 2-Hydroxy-4-(4-nitro-1,3-dioxoisoindolin-2-yl) benzoic acid (IDD-B40).

IDD-B40 and its other 24 derivatives within the library were synthesized at the Korean Chemical Bank of the Korean Research Institute of Chemical Technology. The detailed synthesis procedure of IDD-B40 is described in the supplementary material. However, it was finally collected as an off-white solid with the molecular formula of C_15_H_8_N_2_O_7_ (Fig. 1). The structural and anti-mycobacterial activity information of all 25 isoindole derivatives is provided in supplementary table S1.

### Commercial drugs

Isoniazid (INH), rifampicin (RIF), streptomycin (STR), ethambutol (EMB), pyrazinamide (PZA), vancomycin (VAN), and methicillin (METH) were purchased from Sigma-Aldrich (St Louis, MO, USA).

### *M. tuberculosis* strains

Clinically isolated MDR strains (KMRC 00116-00023, KMRC 00116-00072, KMRC 00116-00082, KMRC 00116-00087, KMRC 00116-00107, KMRC 00116-00111, KMRC 00116-00122, KMRC 00116-00150, KMRC 00116-00181, KMRC 00116-00185, KMRC 00116-00232) and XDR strains (KMRC 00203-00197, KMRC 00203-00052, KMRC 00203-00060, KMRC 00203-00063, KMRC 00203-00085, KMRC 00203-00092, KMRC 00203-00117, KMRC 00203-00128, KMRC 00203-00169, KMRC 00120-00137, KMRC 00121-00341) of *M. tuberculosis* were purchased from the Korean Mycobacterium Resource Center (KMRC, Cheongju, Chungbuk, Korea). *M. tuberculosis* H37Rv (ATCC 27294) was purchased from the American Type Culture Collection (ATCC, Manassas, VA, USA)^9,10^.

### Anti-mycobacterial susceptibility assay

The anti-mycobacterial activities of the test compounds were initially identified by resazurin microtiter assay. Later, we did a luminescent microbial cell viability assay and standard plate count assays for the confirmation.

### Resazurin assay

Resazurin assay was used for the preliminary screening of the anti-mycobacterial activity of IDD-B40 as described previously^9,11^. In short, *Mycobacterium tuberculosis* strains of H37Rv (ATCC 27294) and XDR (KMRC 00203-00197) inocula were prepared in Middlebrook 7H9 broth (BD, USA) and treated with predetermined concentrations of IDD-B40 and control drugs in 96-well plates. After the incubation, 0.2% resazurin solution was added and incubated more until the blue colour changed to pink. Finally, the 50% minimum inhibitory concentration (MIC_50_) was defined as the lowest concentration of a drug that prevented the colour change. The details of the procedure are described in the supplementary materials.

### Luminescent microbial cell viability assay

The number of viable mycobacterial cells was measured in IDD-B40 and control-drugs-treated culture, following the protocol described previously^9,11^, by measuring ATP production using a luminescent viability assay. Briefly, *M. tuberculosis* cultures were treated with predetermined concentrations of drugs in 96-well plates. Following incubation, the drug-treated culture was mixed with freshly prepared BacTiter-Glo reagent, and luminescence was measured with a multilabel reader. The detailed procedure is described in the supplementary materials.

### CFU enumeration assay

A CFU enumeration assay was used to measure the killing effect of IDD-B40 following the previous protocol^9,11^. Briefly, *M. tuberculosis* cultures were treated with predetermined concentrations of drugs in 96-well plates. After incubation, cell suspensions were diluted and spread on agar plates. After three weeks of incubation, colonies were then enumerated. The complete method is provided in the supplementary materials.

### Chequerboard synergy assay

Combinational activities of IDD-B40 with EMB, INH, RIF, and STR were evaluated against *M. tuberculosis*, H37Rv (ATCC 27294) and XDR (KMRC 00203-00197) in 96-well plates using a chequerboard titration method and the experiment was carried out in triplicate^9,11^. Briefly, 96-well plates containing mycobacterial inocula twofold serial dilutions of each drug in Middlebrook 7H9 broth. Following incubation, we calculated fractional inhibitory concentration indices (FICI). The complete procedure is provided in the suplementary material.

### Cytotoxicity test

The cytotoxicity of IDD-B40 was evaluated at a wide range of concentrations (0.02–100 μg/ml) against six different mammalian cell lines (Raw 264.7, L929, A549, HEPG2, Sh-SY5Y, and THP1) by MTT assay^9^. In brief, all the different cells were seeded in 96-well microtiter culture plates and incubated overnight. After reaching 80% confluency, the cells were exposed to test drugs in new culture media for 24 h and had their cytotoxicity evaluated from formazan production. Finally, the selectivity index (SI) was calculated by dividing 50% cytotoxic concentration (CC_50_) by the MIC_50_ of IDD-B40 to check whether it was pharmacologically safe to use or not. All the details are described in the supplementary material.

### Intracellular anti-mycobacterial activity

Intracellular killing activity of IDD-B40 was assessed in Raw 264.7 cell monolayers^9,11^. Briefly, the cell monolayer was grown overnight and infected with both H37Rv (ATCC 27294) and XDR (KMRC 00203–00197. Subsequently, the infected cells were treated with test drugs in fresh culture media for a predetermined time. Then all the cells were lysed to find the number of viable mycobacteria by plating of serially diluted lysates onto agar plates. The entire procedure is provided in the supplementary material.

### Post-antibiotic effect (PAE)

PAEs were determined for the IDD-B40 and control drugs following a modified version of the protocol used in previous articles^9,12^. In short, early log phase *M. tuberculosis* cells were exposed to test drugs in a common concentration of 10 µg/ml, incubated for a short time, and then washed. Finally, the washed cells were incubated until they reached growth saturation (OD_max_), and we calculated their PAE according to Odenholt Tornqvist^12^. The whole process is described in the supplementary material.

### Mycolic acid extraction

We measured the total mycolate from the mycobacterial cell wall treated with the control drug and our test drug to find out whether IDD-B40 had any effect on mycolic acid biosynthesis or not using the acid methanolysis method as previously described^13,14^. Briefly, a liquid culture of mycobacterial cells in the early log phase (OD_600_ 0.2) were treated with the predetermined concentrations of IDD-B40 and INH. After the desired period (7 days) of the treatment, the total mycolic acid of the cells was extracted, and mycolates were separated on the TLC plates. The whole process is provided in the supplementary material.

### RNA isolation and RT-PCR

We used RT-PCR to explore the gene transcript levels of mycolic acid synthesis^10^. We selected 26 genes that were mainly involved in the mycolic acid synthesis, previously described^15^. Briefly, an H37Rv (ATCC 27294) mycobacterial culture was treated with the predetermined concentrations of IDD-B40 and INH. After a desired period of the treatment, we collected RNA samples, reverse transcribed them, and finally did real-time PCR using mycolic acid synthesis gene-specific primers. The details of the used primers and the complete process are given in the supplementary material.

### Evaluation of the activity against clinically significant bacteria

We used a broth microdilution assay to evaluate the effect of IDD-B40 against 24 different clinically significant^9,10^. Briefly, 96-well microplates containing the two-fold dilution of the compound were inoculated. Later, the MIC_50s_ were identified with the lowest concentrations that showed complete inhibition of visible growth. The whole process is provided in the supplementary material.

### Determination of activity against nontuberculous mycobacteria (NTM)

A total of 27 NTM strains were purchased from the Korean Mycobacterium Resource Center (KMRC, Cheongju, Chungbuk, Korea). The activity of IDD-B40 and other control drugs against all the available NTM strains was determined using the broth microdilution technique following the guidelines stated by the Clinical and Laboratory Standards Institute (2015)^9^. The detailed experimental procedure is provided in the supplementary material.

### Statistical analysis

All experiments were carried out in triplicate. Statistical analyses were done using GraphPad Prism 7 software. Means of the drug-free control and drug-treated groups were compared using an unpaired Student's *t* test (*p < 0.05, **p < 0.01, ***p < 0.001, ****p < 0.0001).

### Experimental approval

All the experiments were designed and performed following the protocols and guidelines approved by Soonchunhyang University research quality control committee.

## Results

### Activity of IDD-B40 against drug-susceptible and drug-resistant strains of *M. tuberculosis*

**Figure 2:**
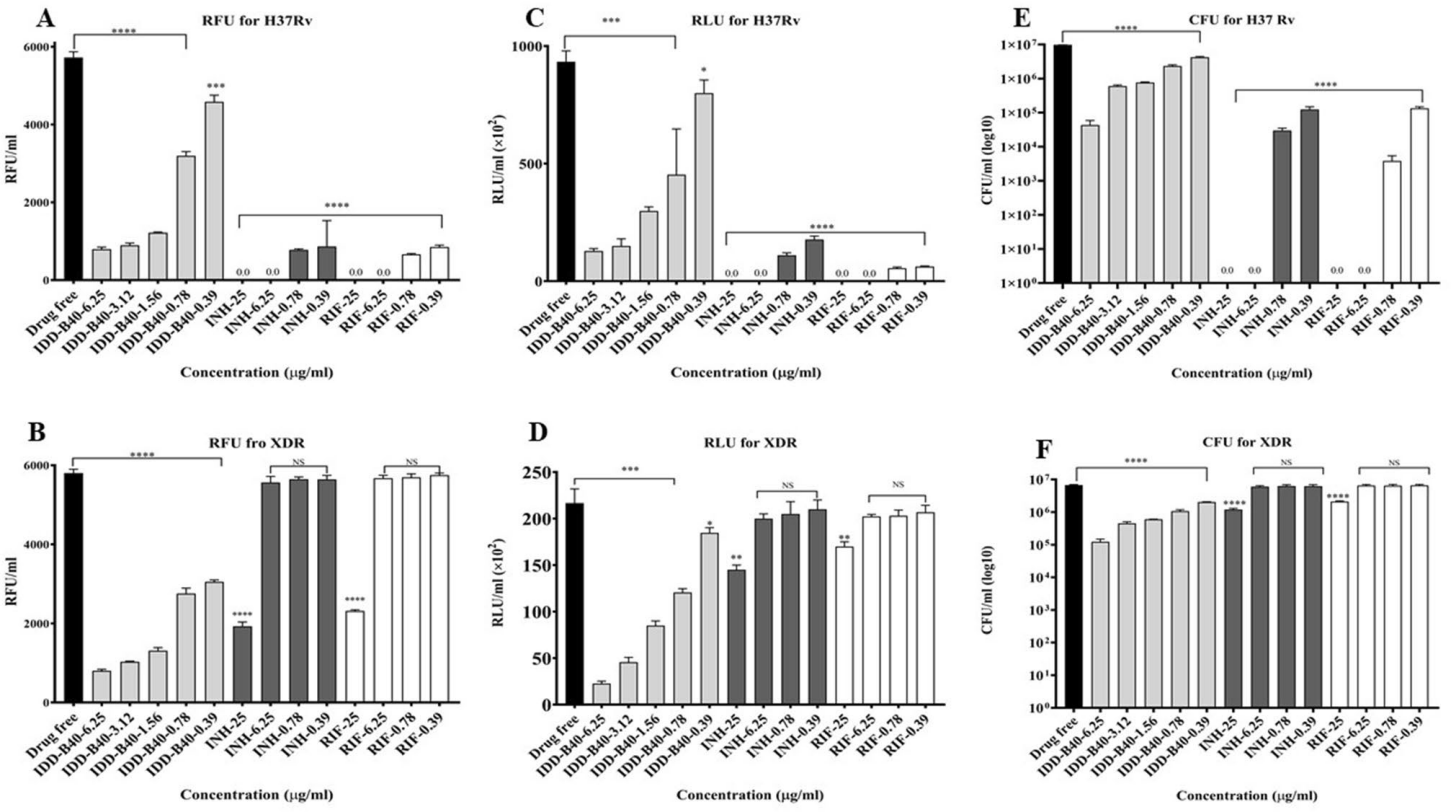
Antimicrobial activities of IDD-B40 and control drugs, including isoniazid (INH) and rifampicin (RIF), against *M. tuberculosis* H37Rv (**A**,**C**,**E**) and extensively drug-resistant (XDR)-*M. tuberculosis* (**B**,**D**,**F**). Mycobacterial susceptibility was assessed by resazurin assay (**A**,**B**) as RFU/ml (relative fluorescence unit per ml), by microbial cell viability assay (**C**,**D**) as RLU/ml (relative luminescence unit per ml), and by CFU enumeration assay (**E**,**F**) as CFU/ml. These experiments were carried out in triplicate. Data are given as mean values and standard deviations. *Statistical significance were determined in Graph Pad Prism 8.2.0 between drug treated *versus* drug-free control using an unpaired Student’s *t *test (*p < 0.05, **p < 0.01, ***p < 0.001, ****p < 0.0001).

The Resazurin assay showed MIC_50s_ of 0.39 μg/ml for IDD-B40 (Fig. 2A,B) against the control *M. tuberculosis* H37Rv (ATCC 27294) and XDR (KMRC 00203-00197). Also, the identical results of 0.39 μg/ml from both the microbial cell viability assay and CFU enumeration assay confirmed the MIC_50s_ for IDD-B40 against the tested control H37Rv and XDR. Altogether, INH and RIF showed a significant effect at lower concentrations against the control H37Rv like IDD-B40. However, INH and RIF required much higher concentration than IDD-B40 against the control XDR (Fig. 2C–F).

**Figure 3:**
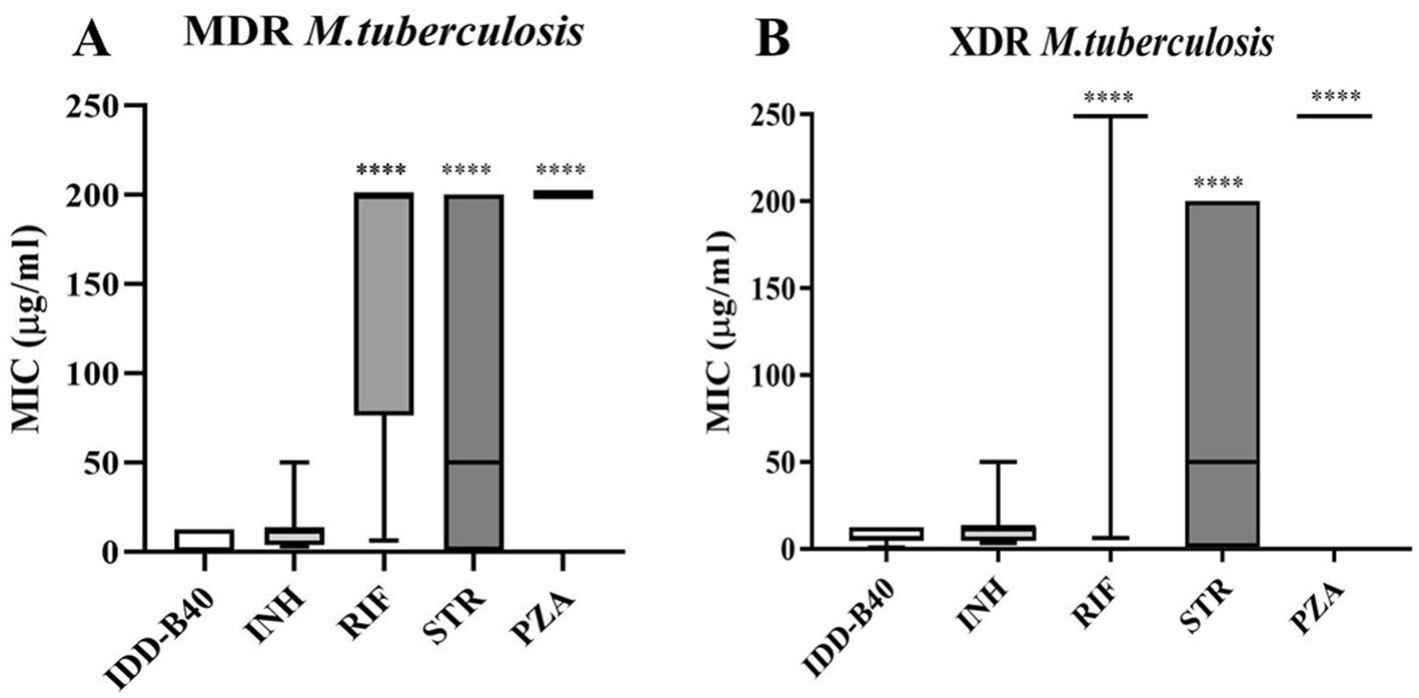
50% Minimum inhibitory concentration (MIC_50_) of IDD-B40 and control drugs [isoniazid (INH), rifampicin (RIF), streptomycin (STR) and pyrazinamide (PZA)] against clinically isolated (**A**) multidrug-resistant (MDR) and (**B**) extensively drug-resistant (XDR) strains of *M. tuberculosis* in Korea. The figures are shown as a box plot, in which the bottom and top edges of the box are the 25th and 75th percentiles, and the center horizontal line is the 50th percentile (median). *Statistical significance were determined in Graph Pad Prism 8.2.0 between IDD-B40 versus control drugs using an unpaired Student’s *t *test (*p < 0.05, **p < 0.01, ***p < 0.001, ****p < 0.0001).

Finally, along with other control drugs (INH, RIF, STR, and PZA), IDD-B40’s activity against 22 additional clinical isolates of MDR and XDR *M. tuberculosis* was further evaluated. In this experiment, IDD-B40 again showed superior activity than the control drugs against all the tested drug-resistant *M. tuberculosis* (MDR and XDR) with MIC_50s_ ranging from 0.02 to 12.5 μg/ml (Fig. 3).

### Antibiotic synergy of IDD-B40

IDD-B40 showed a significant synergistic effect in combination with RIF against both the control H37Rv (ATCC 27294) and XDR (KMRC 00203–00197) *M. tuberculosis*, with the FIC indices of 0.50. In combination with INH and EMB, IDD-B40 showed additive effects against both the tested H37Rv (ATCC 27294) and XDR (KMRC 00203–00197). However, together with STR, IDD-B40 showed a partial synergistic activity against the H37Rv (ATCC 27294) and an additive activity against the XDR (KMRC 00203–00197) (Supplementary Table S2).

### Cytotoxicity of IDD-B40

**Figure 4:**
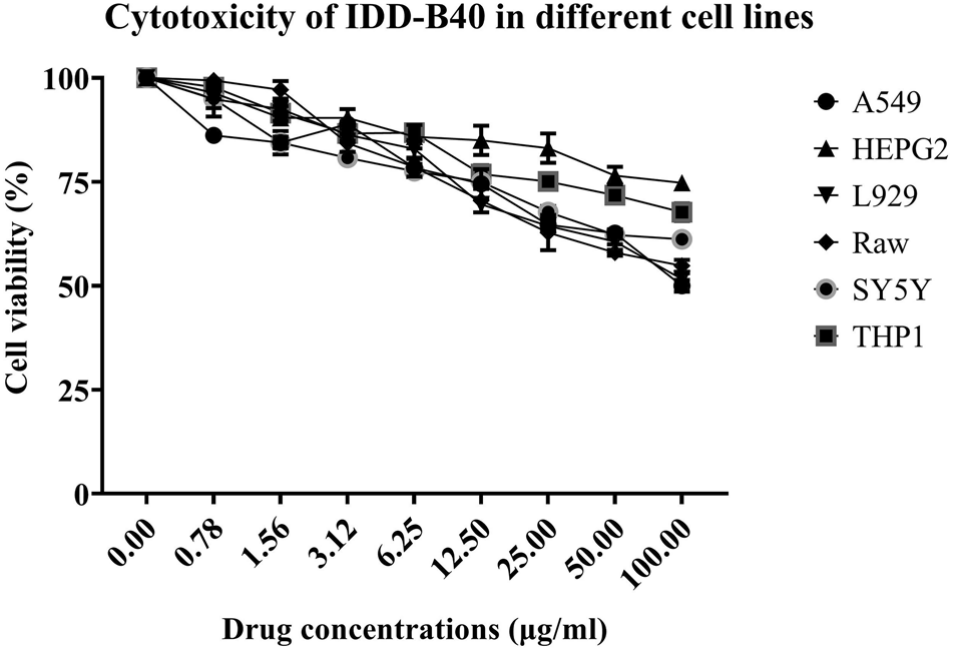
Cytotoxicity of IDD-B40 was evaluated at a wide range of concentrations (100–0.02 μg/ml) against six different mammalian cell lines (Raw 264.7, L929, A549, HEPG2, Sh-SY5Y, and THP1) by MTT assay.

We evaluated the cytotoxicity of IDD-B40 against six different mammalian cell lines (Raw 264.7, L929, A549, HEPG2, Sh-SY5Y, and THP1) by MTT assay. The results indicated that IDD-B40 did not have any significant cytotoxicity up to a concentration of 100 μg/ml and it was safe for further evaluation as the lowest selectivity index, 128.2, among all tested cell lines (Fig. 4 and Supplementary Table S3) was higher than the recommended minimum acceptable limit, 10^16^.

### Intracellular anti-mycobacterial activity of IDD-B40 against *M. tuberculosis* in macrophages

**Figure 5:**
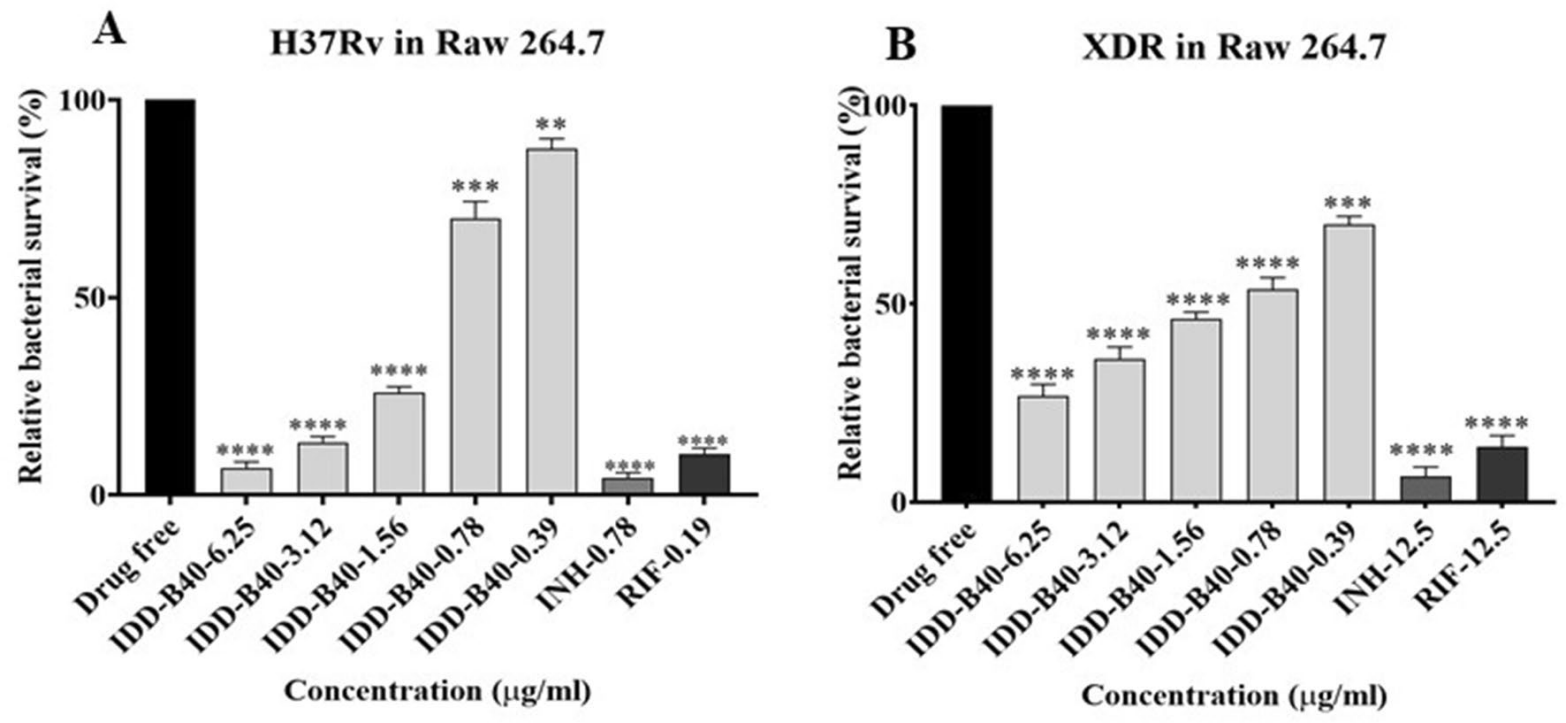
Intracellular killing effect of IDD-B40, isoniazid (INH) and rifampicin (RIF) in *M. tuberculosis*-infected macrophages (Raw 264.7). (**A**) Relative survival of H37Rv in H37Rv-infected macrophages after IDD-B40, INH, and RIF treatment. (**B**) Relative survival of XDR-TB in XDR-TB-infected cells after IDD-B40, INH, and RIF treatment. Data represent the mean ± SD of three independent experiments done in triplicate. ***p < 0.001, ****p < 0.0001 by Student’s *t* test.

IDD-B40 showed a substantial intracellular killing activity against *M. tuberculosis* in a dose-dependent manner. At a concentration of 0.39 μg/ml, IDD-B40 significantly reduced the survival percentage of the infecting H37Rv and XDR *M. tuberculosis* in the Raw 264.7 cell line (Fig. 5). Although the intracellular inhibition activity of IDD-B40 against H37Rv was similar to that of the control drugs: INH and RIF, the intracellular activity of IDD-B40 against the XDR was significantly superior to INH and RIF.

### PAE of IDD-B40 in *M. tuberculosis*

**Figure 6:**
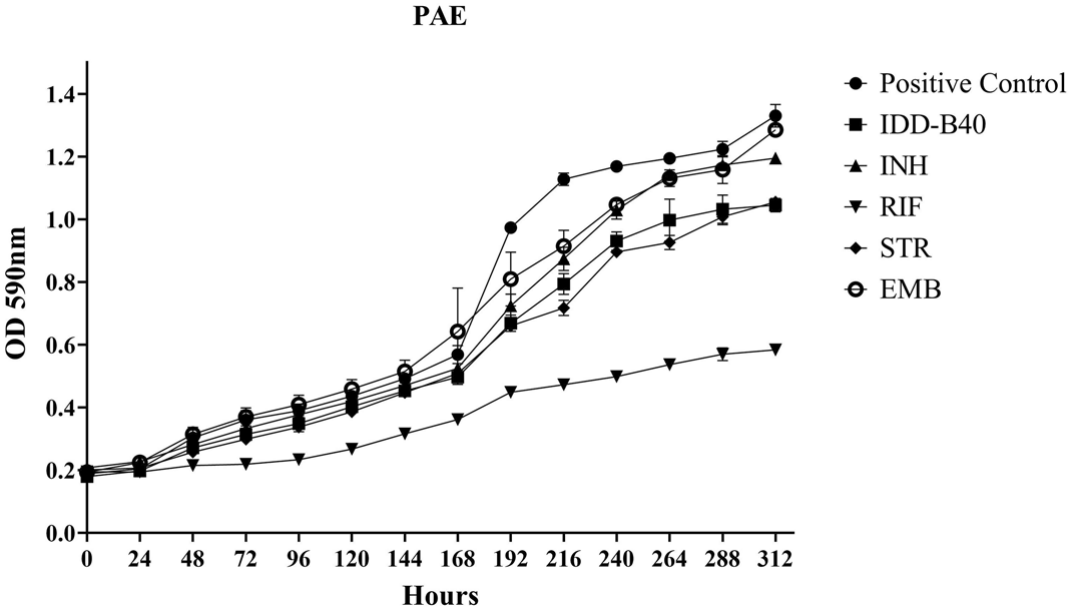
Post-antibiotic effect (PAE) from the growth of *M. tuberculosis* after pulse dosing with IDD-B40, isoniazid (INH), rifampicin (RIF), streptomycin (STR), and ethambutol (EMB) at a concentration of 10 µg/ml. RIF shows the lowest PAE value of 144 h, followed by STR (40 h), IDD-B40 (29 h), EMB (28 h), and INH (20 h).

In this study, we used OD_600_ to find the PAE of IDD-B40 along with first-line control drugs. Following 2 h of pulse exposure to 10 µg/ml each of IDD-B40, INH, RIF, STR, and EMB, the growth of *M. tuberculosis* was retarded, as reflected by the long PAE recovery time (Fig. 6). The PAE value of IDD-B40 was 29 h, which was superior to INH and EMB (PAE values of 20 h, and 28 h, respectively). Only RIF and STR showed a longer PAE value (144 h and 40) than did IDD-B40.

### Activity of IDD-B40 against clinically significant bacteria

IDD-B40 was further tested for its in vitro activity against 24 clinically significant non-mycobacterial pathogens. IDD-B40 showed no activity against any of the tested bacteria at the highest tested concentration (50 μg/ml). However, among the control drugs, RIF showed considerable activity against most of the tested pathogens (Supplementary Table S4).

### Activity of IDD-B40 against nontuberculous mycobacteria (NTM)

The activity of IDD-B40 against 27 additional nontuberculous mycobacteria (NTM) was also evaluated along with the control drugs INH, RIF, and STR. Unlike the intense activity shown against the different strains of *M. tuberculosis*, IDD-B40 showed no significant effect against any of the tested NTM strains (Supplementary Table S5). However, the control drugs INH, RIF, and STR showed a considerable effect against most of the tested NTM strains (Supplementary Table S5).

### Effects of IDD-B40 on mycolic acid biosynthesis in H37Rv

**Figure 7:**
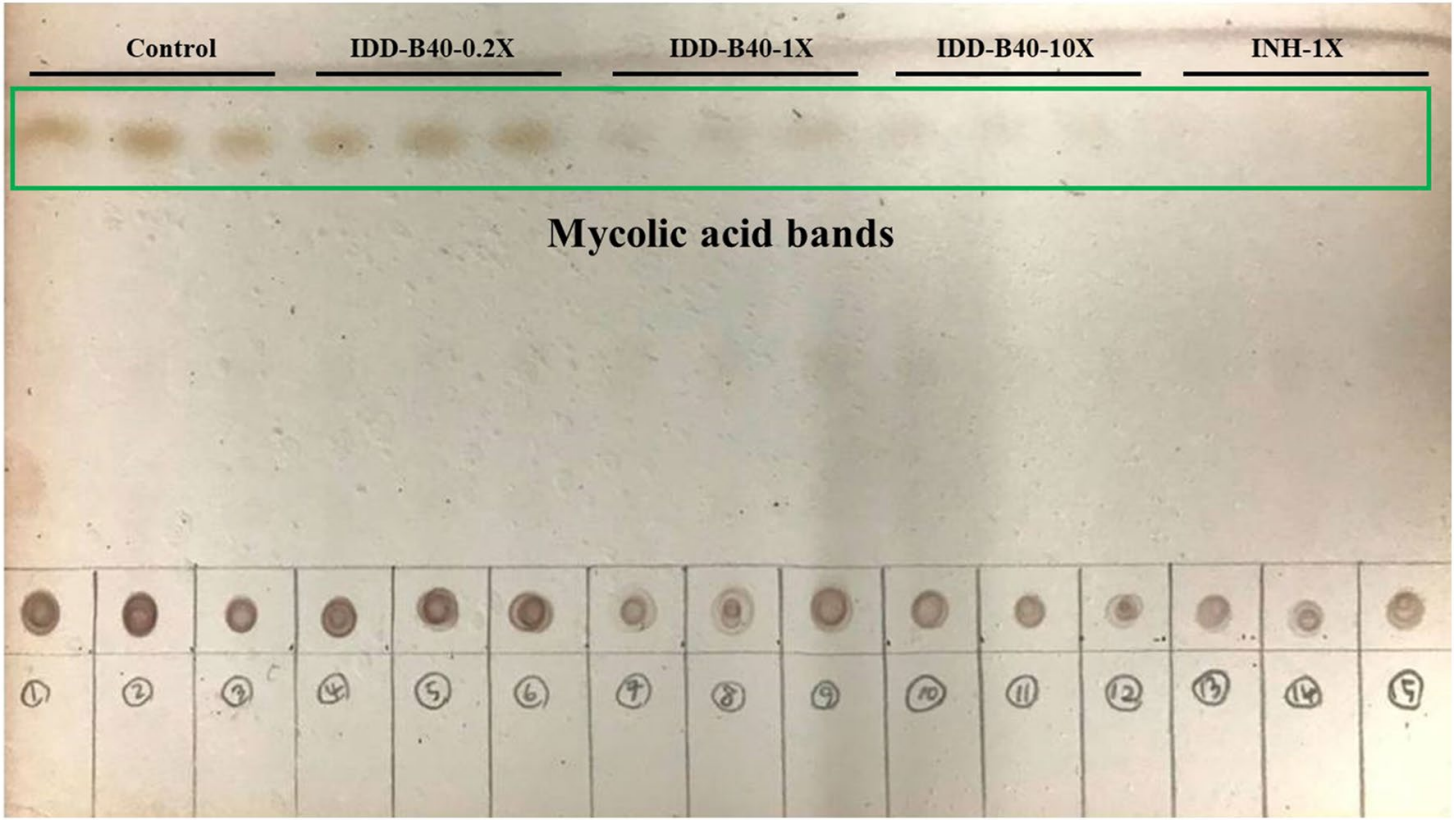
TLC Picture of mycolic acid extracted from H37Rv *M. tuberculosis cells* after the 7 days treatment of different concentration of IDD-B40 and INH.

We treated an H37Rv culture with 0.2 ×, 1 ×, and 10 × the MIC_50_ values of IDD-B40 and 1 × the MIC_50_ value of INH and subjected it to acid hydrolysis and TLC identification of the mycolic acids, as shown in (Fig. 7). The TLC result suggested that IDD-B40 showed a decrease of mycolic acid biosynthesis in a dose-dependent manner of IDD-B40 similar to that of the control mycolic acid inhibitor INH. However, the reduction of mycolic might be due to the killing of whole cells by IDD-B40 rather the inhibition of mycolic acid biosynthesis as IDD-B40 did not have any effect against other mycolic acid-producing bacteria.

### Exploring IDD-B40-induced alterations in mycolic-acid biosynthesis-related gene transcription in *M. tuberculosis*

**Figure 8:**
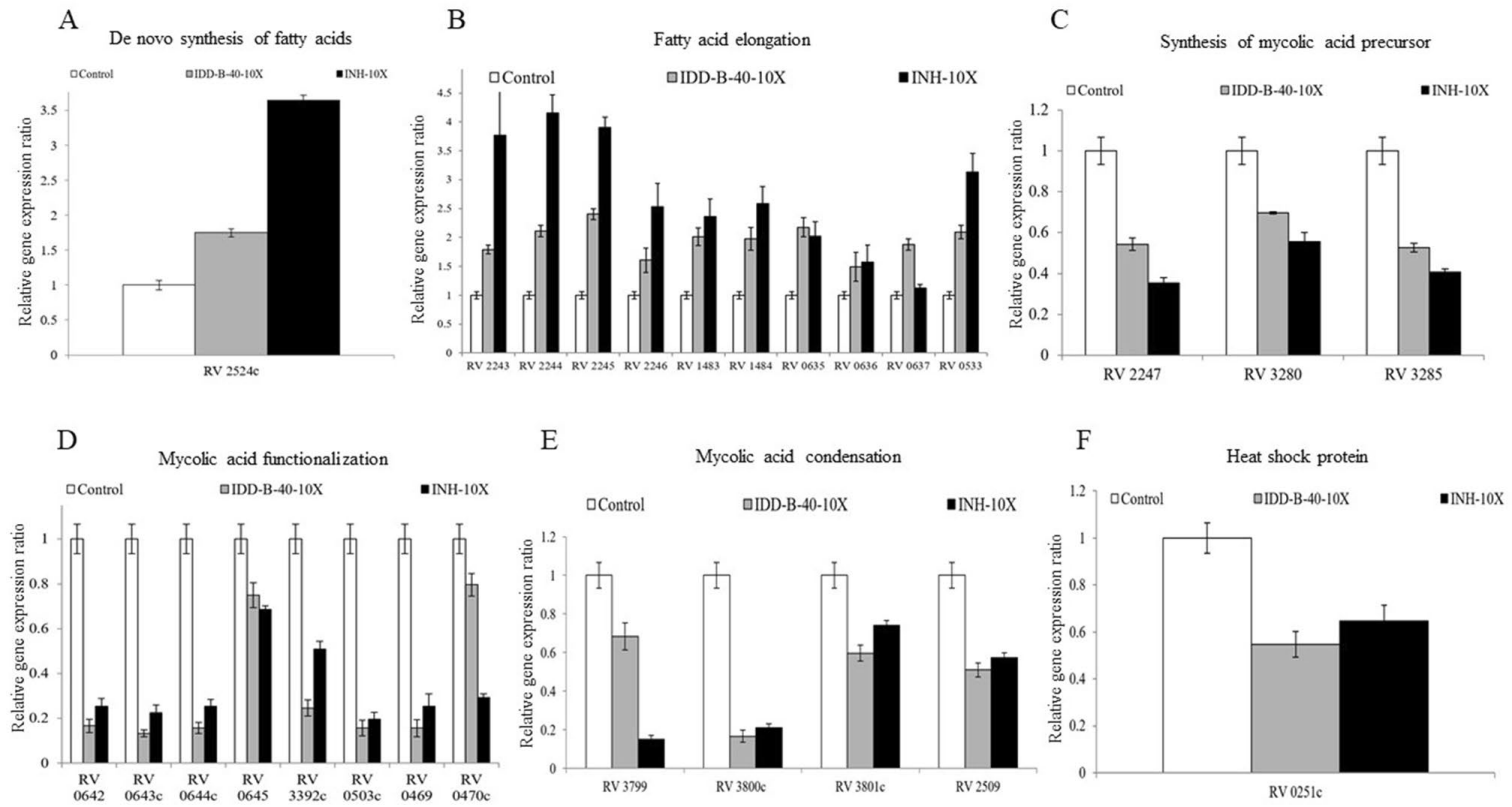
Changes in mRNA levels of *M. tuberculosis* gene expression ratio involved in (**A**–**E**) mycolic acid synthesis and (**F**) heat-shock protein during exposure to 10 × IDD-B40 and INH.

We explored the gene transcript levels of 26 genes related to mycolic-acid synthesis among three groups of samples: control, 10 × MIC_50_ IDD-B40, and 10 × MIC_50_ INH. For the treatment with IDD-B40 and INH, the expression of genes the “de novo synthesis of fatty acids”, “fatty acid elongation” were increased (Fig. 8A,B) compared to control; however, the genes of the “synthesis of mycolic acid precursor”, “meromycolic acid functionalization”, “mycolic acid condensation” and “heat shock protein” groups were decreased (Fig. 8C–F).

## Discussion

In the whole world, approximately 180 people die every day from tuberculosis, and day by day the number of drug-resistant strains of tuberculosis is rapidly increasing^17^. Currently, available drugs for tuberculosis are also losing their activity against newly emerging resistant *M. tuberculosis*^18^. We immediately need new and efficient drugs that can kill these deadly resistant bacteria; otherwise, the whole world will be in great danger eventually.

Many published reports have shown that the indole and isoindole group has a potent anti-tuberculosis activity and could be an efficient candidate for the tuberculosis drug regime^19,20^. In this article, we evaluated the anti-tuberculosis activities of an isoindole based chemical library containing a total of 25 derivatives (Supplementary Table S1). Among all the tested derivatives, IDD-B40, an isoindoledione compound, showed strong activities against *M. tuberculosis* in the preliminary screening and was selected for further advanced studies of drug development.

IDD-B40 showed significant activity not only against drug-sensitive control *M. tuberculosis* (H37Rv, ATCC 27294) but also against control XDR and 22 additional clinical drug-resistant *M. tuberculosis* strains, with MIC_50s_ ranged from 0.02 to 12.5 μg/ml. IDD-B40 showed various MIC_50s_ against different *M. tuberculosis* strains as all the tested strains had different mechanisms of resistance and different origin. Like our findings, Regiane et al. have reported that a new benzoic-acid derivative shows significant activities against different clinical isolates^21^. Additionally, IDD-B40 showed a synergistic effect with RIF against MTB in a chequerboard synergy assay. The strong activity against a significant number of drug-resistant MDR and XDR *M. tuberculosis* and the additional synergy of IDD-B40 with RIF suggest that it might be a potential candidate for the treatment of current tuberculosis.

*M. tuberculosis* can enter into and localize within alveolar macrophages and get protection from some antibiotics^22^. Moreover, for the development of drugs for any intracellular bacteria, one very important factor is whether the candidate drug can work inside the cell or not^22^. Therefore, we evaluated the effect of IDD-B40 in *M. tuberculosis* (H37Rv and XDR) infected Raw 264.7 cells in this study. Our results showed that IDD-B40 had a clear and potent intracellular tuberculosis killing activity, which proved its efficacy as a potential choice for tuberculosis treatment. In addition, IDD-B40 showed no significant toxicity against all six types of mammalian cell lines, and its higher SI indices suggested that IDD-B40 might be pharmacologically safe as a suitable candidate for further evaluation^16^. IDD-B40 demonstrated a better PAE than did INH and EMB. As an extended PAE can allow wider dosing intervals without the loss of therapeutic efficacy, so we can consider IDD-B40 for further study for drug development^23^.

In this study, we further evaluate the activity of IDD-B40 against 24 other clinical pathogens including mycolic acid producing *Corynebacterium diphtheriae*. Finally, we confirmed that IDD-B40 had no activity against non-tubercular pathogens. It showed activity only on *Mycobacterium tuberculosis,* indicating that it is *M. tuberculosis* specific.

We also tried to determine the effect of IDD-B40 on mycolic-acid biosynthesis to discern whether IDD-B40 acted on mycolic acid or not, since mycolic acid is a cell-wall component of mycobacteria and is known to be very important in the physiology of mycobacteria^24^. We checked the changes of mycolic acid-producing genes and the amount of total mycolic acid after the treatment of IDD-B40 and the control mycolic acid inhibitor INH^15,25^. The initial results of these experiments indicated that IDD-B40 might kill *M. tuberculosis* by inhibiting mycolic acid synthesis as both IDD-B40 and INH showed a similar pattern in their results. However, the intense activity of IDD-B40 against INH resistant XDR and MDR *M. tuberculosis* strains and the non-functional properties against mycolic acid-containing *Corynebacterium diphtheriae* together contradicted its probability of mycolic acid inhibition. Finally, the non-inhibitory result of IDD-B40 against the other mycolic acid-producing nontuberculous mycobacterial strains (NTM) strains confirmed that IDD-B40 had a different mode of action rather than mycolic acid inhibition. Also, this result suggested that IDD-B40 was specifically active against *M. tuberculosis.* This type of high specificity is rare as most of the anti-tuberculosis drugs share their activity against other NTM or at least against *M. bovis*^9,26–29^. Although the reason for this high selectivity is not clear, the noble mode of action of IDD-B40 might play the role behind this but, it is yet to be discovered.

## Conclusions

In summary, IDD-B40 demonstrates strong activity against both drug-susceptible and drug-resistant strains of *Mycobacterium tuberculosis.* It also exhibits direct synergy against both the control H37Rv and XDR in combination with rifampicin and shows no significant cytotoxicity to any of the tested cell lines. However, it is still not clear why IDD-B40 was highly selective against *M. tuberculosis* and showed an INH like result in the mycolic acid gene expression experiment. The investigation of these queries and the evaluation of IDD-B40’s efficacy in an animal model is further required to carry out the next phase of experiments necessary for new drug development.

## Supplementary Information

Supplementary Information.
