# Supplementary material for: Antimicrobial activity of IDD-B40 against drug-resistant Mycobacterium tuberculosis
Source: Sci Rep. 2021 Jan 12;11:740. doi: 10.1038/s41598-020-80227-y (PMC7804135; doi:10.1038/s41598-020-80227-y)
Supplement: Supplementary file 1 — Supplementary Information. [file 41598_2020_80227_MOESM1_ESM.docx]

**Antimicrobial activity of IDD-B40 against drug-resistant *Mycobacterium tuberculosis***

Md Imtiazul Islam^1^, Hoonhee Seo^2^, Sukyung Kim^2^, Venkata S. Sadu^3^, Kee-In Lee^3,4^ and Ho-Yeon Song^1,2^*

Department of Microbiology and Immunology, School of Medicine, Soonchunhyang University, Cheonan, Chungnam, 31151, Korea^1^

Probiotics Microbiome Convergence Center, Soonchunhyang University, Asan, Chungnam, 31538, Korea^2^

Green Chemistry Division, Korea Research Institute of Chemical Technology, Daejeon 34114, South Korea^3^

Major of Green Chemistry and Environmental Biotechnology, University of Science & Technology, Daejeon 34113, South Korea^4^

Md Imtiazul Islam and Hoonhee Seo contributed equally to this study.

Corresponding Author, Ho-Yeon Song, MD, PhD

Department of Microbiology, College of Medicine, Soonchunhyang University, Cheonan, Chungnam 31151, South Korea

Tell: +82-41-570-2412, Fax: +041-577-2415

**E-mail address:** songmic@sch.ac.kr

**Supplementary Information**

**Materials and methods**

***Isoindoledione derivatives***

Initially, 25 isoindoledione derivatives were synthesized at the Korean Chemical Bank of the Korean Research Institute of Chemical Technology, Korea.

**Supplementary Table S1.** Structures of isoindoledione derivatives and their *in vitro* anti-tubercular activities against *M. tuberculosis* H37Ra and H37Rv.

| IDD- | R^1^ | R^2^ | R^3^ | MIC (µg/ml) | |
| --- | --- | --- | --- | --- | --- |
|  |  |  |  | H37Ra | H37Rv |
| B39 | H | OH | H | >50 | >50 |
| B40 | H | OH | COOH | 0.39 | 0.39 |
| B42 | H | OH | CO_2_Me | >50 | >50 |
| B67 | OH | H | Me | >50 | >50 |

| IDD- | R^1^ | R^2^ | R^3^ | MIC (µg/ml) | |
| --- | --- | --- | --- | --- | --- |
|  |  |  |  | H37Ra | H37Rv |
| B44 | OH | OH | H | >50 | >50 |
| B45 | H | OH | OH | >50 | >50 |
| B46 | H | OMe | OMe | >50 | >50 |
| B51 | OH | OMe | H | >50 | >50 |

| IDD- | R^1^ | R^2^ | R^3^ | MIC (µg/ml) | |
| --- | --- | --- | --- | --- | --- |
|  |  |  |  | H37Ra | H37Rv |
| B47 | H | Me |  | >50 | >50 |
| B50 | NO_2_ | H |  | >50 | >50 |
| B61 | H | H |  | >50 | >50 |
| B62 | H | H |  | >50 | >50 |
| B66 | H | Me |  | 25 | 25 |

| IDD- | MIC (µg/ml) | |
| --- | --- | --- |
|  | H37Ra | H37Rv |
| B52 | >50 | >50 |

| IDD- | MIC (µg/ml) | |
| --- | --- | --- |
|  | H37Ra | H37Rv |
| B59 | >50 | >50 |

| IDD- | R | MIC (µg/ml) | |
| --- | --- | --- | --- |
|  |  | H37Ra | H37Rv |
| B60 |  | >50 | >50 |
| B64 |  | >50 | >50 |
| B65 |  | >50 | >50 |
| B75 |  | >50 | >50 |

| IDD- | X | R^1^ | R^2^ | R^3^ | MIC (µg/ml) | |
| --- | --- | --- | --- | --- | --- | --- |
|  |  |  |  |  | H37Ra | H37Rv |
| B68 | N | H | H | H | >50 | >50 |
| B70 | CH | H | Me | H | >50 | >50 |
| B71 | CH | OH | OH | H | >50 | >50 |
| B72 | CH | H | Cl | H | >50 | >50 |
| B73 | N | H | H | Br | >50 | >50 |
| B74 | CH | Cl | NO_2_ | H | >50 | >50 |

***Synthesis of 2-Hydroxy-4-(4-nitro-1,3-dioxoisoindolin-2-yl) benzoic acid (IDD-B40)***

To a suspension of 3-nitrophthalic acid (5.0 g, 23.7 mmol) in Toluene (50 mL), oxalyl chloride (2.93 g, 29.6 mmol) was added dropwise over 10 minutes at room temperature under Argon atmosphere. After refluxing for 3 h, the solvent and excess oxalyl chloride were removed under vacuum. The resultant residue was dissolved in CH_3_COOH (30 mL), 4-amino-2-hydroxybenzoic acid (4-aminosalicylic acid) (3.0 g, 19.66 mmol) was added, and it was refluxed for 4 hours. The reaction mixture was cooled to room temperature, and the precipitated solid was filtered, washed with CH_3_COOH (10 mL), and water (50 mL), and dried under vacuum to afford 2-hydroxy-4-(4-nitro-1,3-dioxoisoindolin-2-yl)benzoic acid (3.0 g, 39%) as an off-white solid;^1^H NMR (500 MHz, DMSO): *δ*_H_ 11.45 (s, 1H), 8.40 - 8.33 (m, 1H), 8.28 (d, *J* = 7.3 Hz, 1H), 8.14 (t, *J* = 7.8 Hz, 1H), 7.94 (d, *J* = 8.4 Hz, 1H), 7.10 (d, *J* = 1.9 Hz, 1H), 7.05 (dd, *J* = 8.4, 1.9 Hz, 1H) ppm; ^13^C NMR (126 MHz, DMSO): *δ*_C_ 171.7, 165.2, 162.6, 161.4, 145.0, 137.8, 136.9, 133.9, 131.2, 128.9, 127.6, 123.3, 118.4, 116.1 ppm; HRMS-EI *m/z* [M]^+^ calcd for C_15_H_8_N_2_O_7_, 328..0337, found 328.0332 (Figure 1).

**Supplementary Fig. S1.** Synthetic scheme of IDD-B40

*
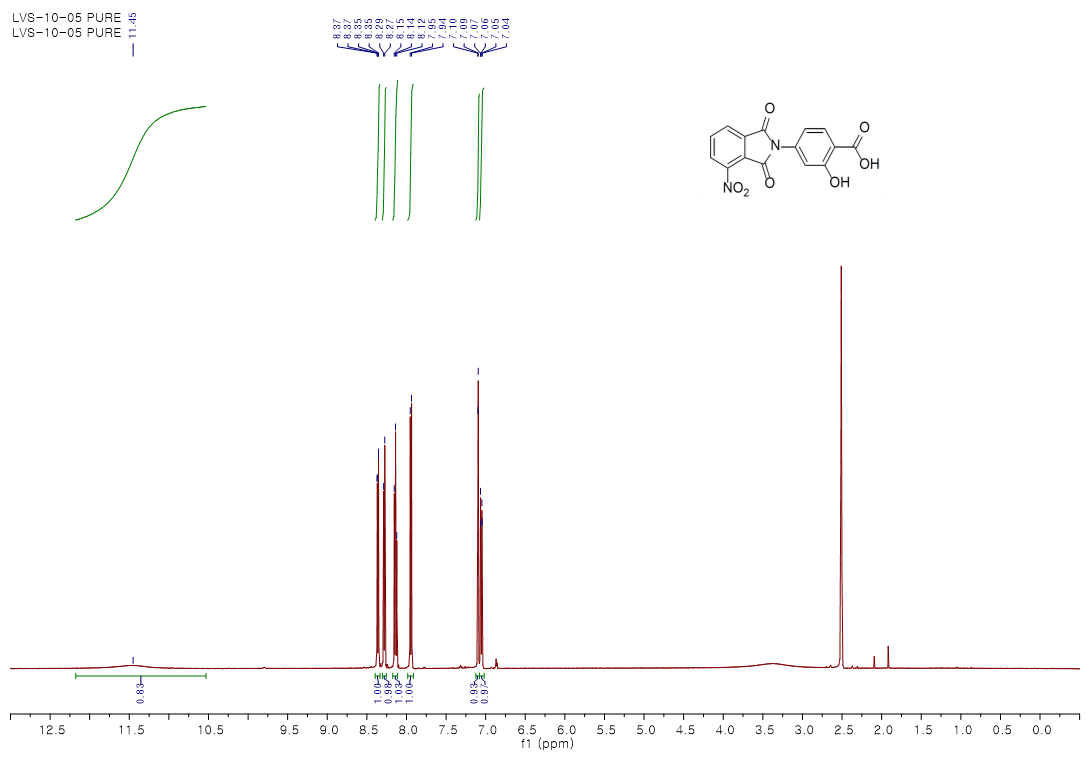
*

**Supplementary Fig. S2.** ^1^H-NMR of IDD-B40 for its structural assignment

*
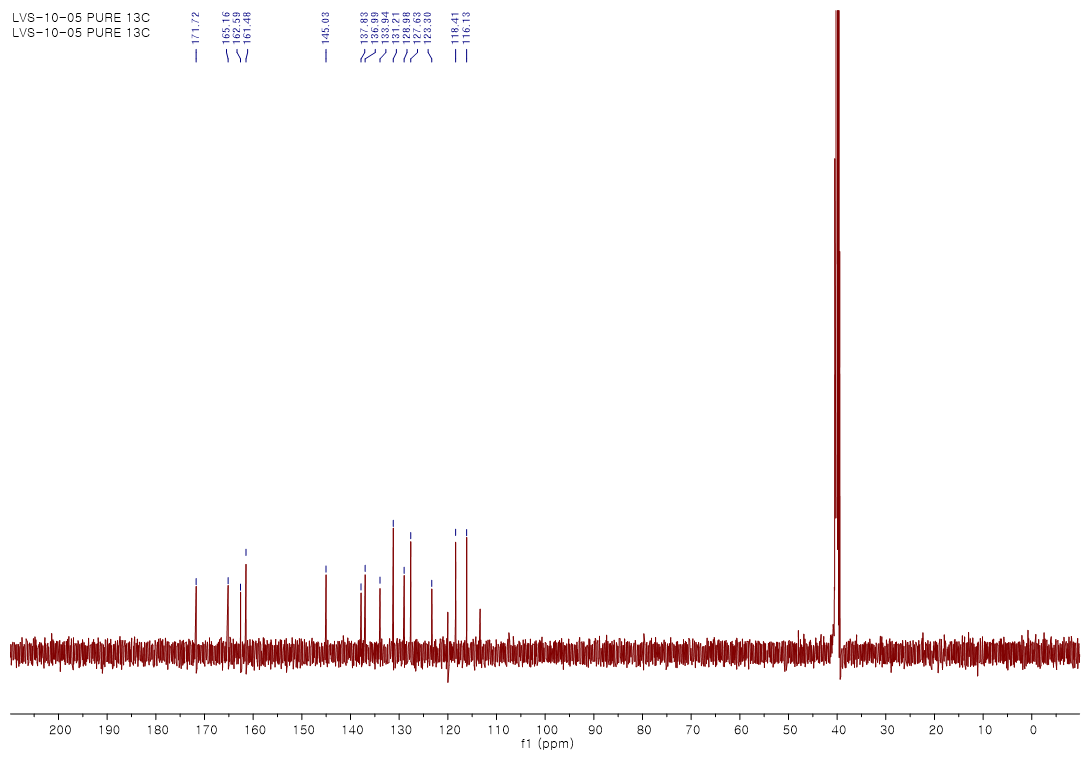
*

**Supplementary Fig. S3.** ^13^C-NMR of IDD-B40 for its structural assignment

***Anti-mycobacterial susceptibility assay***

***Resazurin assay***

The whole process was previously described. ^1,2^ In 96-well plates, *Mycobacterium tuberculosis* strains of H37Rv (ATCC 27294) and XDR (KMRC 00203-00197) inocula were prepared in Middlebrook 7H9 broth (BD, USA) supplemented with 10% albumin dextrose catalase (ADC) (BD, USA) and 0.2% glycerol to a final density of 1 ×10^5^ cfu/ml in 200 µl containing predetermined concentrations of IDD-B40 and control drugs (200-0.02 µg/ml). Without any drug, the same concentration of bacterial cells with solvent (DMSO) was used as a positive control, and without any bacteria and drugs only fresh media was used as the negative control. After 7 days of incubation at 37 °C, 20 µl of freshly prepared 0.2% resazurin solution was added to each well and made 0.02% final concentration of resazurin, incubated up to 48 hours more until the blue color changed to pink. After additional incubation, the color changes were assessed, and fluorescence readings were taken at 570 and 600 nm with a multilabel reader (Perkin Elmer Victor X3). Minimum inhibitory concentration (MIC) was defined as the lowest concentration of a drug that prevented color change.

***Luminescent microbial cell viability assay and CFU enumeration assay***

The viable mycobacterial cells of H37Rv (ATCC 27294) and XDR (KMRC 00203-00197) in IDD-B40 and control drug-treated culture were enumerated by using a luminescent viability assay kit (G8231, Promega) according to the manufacturer's instructions and previous articles. ^1, 2^ In 96-well plates, bacterial inocula were inoculated in Middlebrook 7H9 broth (BD, USA) supplemented with 10% albumin dextrose catalase (ADC) (BD, USA) and 0.2% glycerol to a final density of 1 ×10^5^ cfu/ml in 200 µl containing a predetermined concentration of test drugs (200-0.02 µg/ml) and were incubated at 37 °C for 7 days. Without any drugs, the same concentration of bacterial cells with solvent (DMSO) was used as a positive control, and without any bacteria and drugs only fresh media was used as the negative control. After incubation, 50 µl MTB cells were collected from each well, thoroughly resuspended in 50 µl freshly prepared BacTiter-Glo reagent, and incubated at room temperature for 10 min on an orbital shaker. Following incubation, luminescence was measured with a multilabel reader (Perkin Elmer Victor X3). Simultaneously, remaining cell suspensions were diluted in Middlebrook 7H9 broth (BD, USA) and aliquots were spread onto Middlebrook 7H10 (BD, USA) agar plates. After 3 weeks of incubation at 37 °C, colonies were then enumerated.

***Chequerboard synergy assay***

This experiment was designed according to previous articles. ^1, 2^ In 96-well plates, 1 ×10^5^ cfu/ml concentrations of H37Rv (ATCC 27294) and XDR (KMRC 00203-00197) inocula were prepared in 200 µl volumes of Middlebrook 7H9 broth (BD, USA) that contained 2-fold serial dilutions of each drug (IDD-B40, INH, RIF, and STR) either alone or in combination and were incubated at 37 °C. Without any drug, the same concentration of bacterial cells with solvent (DMSO) was used as a positive control, and without any bacteria and drugs only fresh media was used as the negative control. Following 7 days of incubation, 20 µl of freshly prepared 0.2% resazurin solution was added to each well to make a 0.02% final concentration of resazurin; it was incubated up to 48 hours more until the blue color changed to pink. The experiment was performed in triplicate. Color changes were assessed and fractional inhibitory concentration indices (FICI) were calculated using the following formula:

$$FICI=\frac{MIC (antibiotic combined with compound)}{MIC (antibiotic alone)}+\frac{MIC (compound combined with \text{antibiotic})}{MIC (compound alone)}$$

Results of FIC index were interpreted as follows: ≤ 0.5, synergy; > 0.5 to 0.75, partial synergy; > 0.75 to 1.0, additive effect; > 1.0 to 4.0, indifference; and > 4.0, antagonism.

**Supplementary Table S2:** Effectiveness of IDD-B40 in combination with first-line antitubercular drugs

| Organism | Antibiotic combination | FIC_A_ | FIC_B_ | FIC index = FIC_A_ + FIC_B_ | Outcome |
| --- | --- | --- | --- | --- | --- |
| H37Rv | IDD-B40 +RIF | FIC _IDD-B40_ =0.25 | FIC_RIF_=0.25 | 0.5 | Synergy |
|  | IDD-B40 +INH | FIC _IDD-B40_ =0.5 | FIC_INH_= 0.5 | 1.0 | Addagainsttitive effect |
|  | IDD-B40 +STR | FIC _IDD-B40_ =0.25 | FIC_STR_= 0.5 | 0.75 | Partial effect |
|  | IDD-B40 + EMB | FIC _IDD-B40_ =0.5 | FIC_EMB_ = 0.5 | 1.0 | Additive effect |
| XDR | IDD-B40 +RIF | FIC _IDD-B40_ =0.25 | FIC_RIF_=0.25 | 0.5 | Synergy |
|  | IDD-B40 +INH | FIC _IDD-B40_ =0.5 | FIC_INH_= 0.5 | 1.0 | Additive effect |
|  | IDD-B40 +STR | FIC _IDD-B40_ =0.5 | FIC_STR_= 0.5 | 1.0 | Additive effect |
|  | IDD-B40 + EMB | FIC _IDD-B40_ =0.5 | FIC_EMB_ = 0.5 | 1.0 | Additive effect |

INH, isoniazid; RIF, rifampicin; STR, streptomycin; MIC, minimum inhibitory concentration;

FIC, fractional inhibitory concentration; FICI, fractional inhibitory concentration index.

Combinations were tested against *M. tuberculosis* H37Rv and XDR by resazurin microtiter assay.

FIC of drug A = MIC of drug A in combination ÷ MIC of drug A alone;

FIC of drug B = MIC of drug B in combination ÷ MIC of drug B alone.

FICI = FIC of drug A + FIC of drug B.

Results of FIC index were interpreted as follows: ≤ 0.5, synergy; > 0.5 to 0.75, partial synergy; > 0.75 to 1.0, additive effect; > 1.0 to 4.0, indifference; and > 4.0, antagonism.

***Cytotoxicity test***

Cytotoxicity of IDD-B40 was determined following the previously used method. ^2^ Murine Raw 264.7 macrophages (KCLB 40071), L929 murine fibroblasts (KCLB 10001), A549 human pulmonary epithelial (KCLB 10185), HEPG2 (KCLB 88065), SH-SY5Y (KCLB 22266), and THP1 (KCLB 40202) cell lines were purchased from the Korean Cell Line Bank (KCLB) (Seoul, Korea). At first, all six different cells were seeded at a density of 1 ×10^4^ cells in 96-well microtiter culture plates in a 200 µl culture and incubated overnight at 37 °C in a CO_2_ incubator. Specifically, the THP-1 cells were differentiated from monocyte to macrophage using the treatment of 200 nM PMA (Phorbol-12-myristate-13-acetate). In After reaching 80% confluency, the cells were washed with PBS and treated with various concentrations of IDD-B40 (100–0.02 µg/ml) containing media. Without any drug, the same concentration of cells with solvent (DMSO) was used as a positive control, and only fresh media were used as negative control. After 24 hours of incubation following the drug treatment, 20 µl of 0.5 mg/ml 3-[4, 5-Dimethylthiazol-2-yl]-2,5 diphenyltetrazolium bromide (MTT reagent, Sigma) was added into each well, and it was incubated in a CO_2_ incubator at 37 °C for 4 hours. After 4 hours, the medium was aspirated, and the formazan product was solubilized with 100 ul dimethyl sulfoxide (DMSO). Finally, the absorbance was measured at 570 nm with a multilabel reader (Perkin Elmer Victor X3), and 50% cytotoxic concentration (the concentration of drug that kills 50% of viable cells) of IDD-B40 was calculated by using the following formula:

Absorbance of sample - absorbance of blank

% of viable cells = ×100

Absorbance of positive control - absorbance of blank

After getting the 50% cytotoxicity concentration (CC50), we calculated the selectivity indices (SI) of IDD-B40 by using the following formula:

50% cytotoxic concentration (CC_50_) of cell

SI index =

MIC of Drug

**Supplementary Table S3:** Cytotoxicity testing results of IDD-B40

| Cell line | IC50 (µg/ml) | MIC (µg/ml) | SI |
| --- | --- | --- | --- |
| Raw 264.7 | >100 | 0.39 | >128.2 |
| L929 | >100 | 0.39 | >128.2 |
| A549 | 100 | 0.39 | 128.2 |
| HEPG2 | >100 | 0.39 | >128.2 |
| SH-SY5Y | >100 | 0.39 | >128.2 |
| THP1 | >100 | 0.39 | >128.2 |

***Intracellular anti-mycobacterial activity***

Intracellular killing activity of IDD-B40 was assessed in Raw 264.7 cell monolayers following the previous protocol. ^1,2^ A RAW 264.7 cell monolayer (1 ×10^6^ cells/ml) was grown overnight and infected with both H37Rv (ATCC 27294) and XDR (KMRC 00203-00197) by exposing the cell to a multiplicity of infection (MOI) of 10:1 bacilli per cell in 96-well plates for 2 hrs at 37 °C in a CO_2_ incubator to allow uptake of MTB bacilli by the cells. Subsequently, cells were washed with DMEM media to remove the remaining extracellular MTB bacilli, and later the infected cells were cultured in 200 µl of medium in the presence of test drugs for 3 days. Infected cells and solvent (DMSO) in the absence of any test drugs were used as a positive control, and non-infected cells were used as a negative control. After 3 days of drug treatment, infected cells were lysed in 0.2% Triton X-100 for 20 min at 37 °C, and the number of viable bacteria was found by plating of serially diluted lysates onto Middlebrook 7H10 (BD, USA) agar plates.

***Post-antibiotic effect (PAE)***

This experiment was designed according to previous articles. ^2, 3^ In Middlebrook 7H9 broth (BD, USA), H37Rv (ATCC 27294) at early log phase (OD_600_ 0.2) were exposed to IDD-B40, RIF, INH, STR, or EMB at the same concentration of 10 µg/ml for 2 hours. The same concentration of bacterial cells with only solvent (DMSO) was used as a positive control, and without any bacteria and drugs only fresh media were used as a negative control. After 2 hours of drug treatment at 37°C, antibiotics were removed by centrifuging (3600 g, 10 mins) and cell pellets were washed three times in pre-warmed fresh Middlebrook 7H9 broth. Finally, washed pellets were resuspended in a pre-warmed Middlebrook 7H9 broth and incubated at 37 °C until they reached growth saturation (OD_max_). The OD_600_ was calculated for each culture before drug exposure, after drug removal, and at 24-hr intervals thereafter. The duration of PAE was calculated as the time taken for the antibiotic-treated culture to reach 50% of OD_max_ of drug-free culture minus the time taken for the drug-free control to reach the same point.

***Assessment of activity against clinically significant bacteria***

This experiment was designed according to previous articles. ^2, 4^ In 96-well plates, all the tested 24 bacteria in a final concentration of 1 ×10^5^ cfu/ml were inoculated in cation-adjusted Muller Hilton broth (CAMHB, Sigma) containing a predetermined concentration (200-0.02 µg/ml) of tested drugs and were incubated at 37 °C for 18-24 hours. For *Corynebacterium* spp. and *Streptococcus* spp., we added an extra 3% lysed horse blood (LHB) to CAMHB. Only bacteria containing media with solvent (DMSO) and only fresh media were used as positive and negative control, respectively. After incubation, the lowest concentration of drug that inhibited the visual bacterial growth was defined as the MIC of the tested drug.

**Supplementary Table S4:** MIC of IDD-B40and five control drugs against 24 clinically significant

bacterial strains

|  | MIC values (μg/ml) | | | | | |
| --- | --- | --- | --- | --- | --- | --- |
| Strains | IDD-B40 | INH | RIF | STR | VAN | MET |
| *Acinetobacter baumannii* | >50 | >50 | 3-6 | 6-12 | >50 | 3.12-6.25 |
| *Citrobacter freundii* | >50 | >50 | >50 | 1.5-3 | >50 | >50 |
| *Enterobacter aerogenes* | >50 | >50 | 6-12 | 3-6 | >50 | >50 |
| *Escherichia coli* | >50 | >50 | 6-12 | >25 | >50 | >50 |
| *Escherichia coli* O157 | >50 | >50 | 6-12 | 6-12 | >50 | >50 |
| *Klebsiella pneumoniae* | >50 | >50 | 6-12 | 3-6 | >50 | >50 |
| *Proteus mirabilis* | >50 | >50 | 3-6 | 6-12 | >50 | >50 |
| *Proteus vulgaris* | >50 | >50 | 6-12 | 25 | >50 | >50 |
| *Pseudomonas aeruginosa* | >50 | >50 | 24 | >25 | >50 | >50 |
| *Salmonella enteritidis* | >50 | >50 | 12-24 | 3-6 | >50 | >50 |
| *Salmonella paratyphi* A | >50 | >50 | 12-24 | 6-12 | >50 | >50 |
| *Salmonella typhimurium* | >50 | >50 | 12-24 | >25 | >50 | >50 |
| *Serratia marcescens* | >50 | >50 | 12-24 | >25 | >50 | >50 |
| *Shigella boydii* | >50 | >50 | 0.4-0.8 | >25 | 12.5-25 | >50 |
| *Shigella flexneri* | >50 | >50 | 1.5-3 | >25 | >50 | >50 |
| *Shigella sonnei* | >50 | >50 | 3-6 | 3-6 | >50 | >50 |
| *Shigella dysenteriae* | >50 | >50 | 1.5-3 | >25 | >50 | >50 |
| *Staphylococcus aureus* | >50 | >50 | < 0.1 | 6-12 | 1.5-3 | 1.56 |
| *Staphylococcus aureus* MRSA | >50 | >50 | < 0.1 | 3-6 | 0.8-1.5 | >50 |
| *Staphylococcus epidermidis* | >50 | >50 | < 0.1 | 1.5-3 | 0.8-1.5 | >50 |
| *Corynebacterium diphtheriae* | >50 | >50 | < 0.1 | 3-6 | 0.8-1.5 | >50 |
| *Streptococcus pneumoniae* | >50 | >50 | < 0.1 | 12-24 | 0.8-1.5 | 1.5-3 |
| *Streptococcus pyogenes* | >50 | >50 | < 0.1 | 12-24 | 0.1 | 0.4-0.8 |
| *Streptococcus sanguinis* | >50 | >50 | < 0.1 | 12-24 | 0.4-0.8 | 0.8-1.6 |

***Determination of activity against nontuberculous mycobacteria (NTM)***

In 96-well plates, all 27 NTM strains (1 ×10^5^ cfu/ml) were inoculated in 200 µl of Middlebrook 7H9 broth (BD, USA) containing a predetermined concentration of IDD-B40 and control drugs (200-0.02 µg/ml) and incubated at 37 °C for 1 to 7 days according to their doubling time. Only bacteria containing media with solvent (DMSO) and only fresh media were used as positive and negative control, respectively. After incubation, the lowest drug concentration that inhibited visual bacterial growth was determined as the MIC of the tested drug.

**Supplementary Table S5:** MIC of IDD-B40and five control drugs against 24 clinically significant

|  | MIC (μg/ml) | | | |
| --- | --- | --- | --- | --- |
| Strains | IDD-B40 | INH | RIF | STR |
| *M. abscessus* | >50 | >50 | >50 | 25 |
| *M. avium* | >50 | 25 | 1.56 | 3.13 |
| *M. fortuitum* | >50 | 25 | 0.19 | 6.25 |
| *M. intracellulare* | >50 | 25 | <0.09 | 0.78 |
| *M. marinum* | >50 | 25 | <0.09 | <0.09 |
| *M. phlei* | >50 | >50 | 25 | 0.19 |
| *M. szulgai* | >50 | 1.56 | <0.09 | 0.19 |
| *M. xenopi* | >50 | 1.56 | 0.19 | 0.19 |
| *M. gilvum* | >50 | 12.5 | 0.19 | 0.19 |
| *M. smegmatis* | >50 | 25 | 25 | 0.19 |
| *M. bovis* | >50 | 0.19 | <0.09 | 0.19 |
| *M. kansasii* | 25 | 0.19 | 0.04 | 0.19 |
| *M. arupense* | >50 | 50 | 0.1 | 0.1 |
| *M. aubagnense* | >50 | >50 | 1.56 | >50 |
| *M. bolletti* | >50 | >50 | >50 | 25 |
| *M. colombiense* | >50 | 12.5 | 0.19 | 0.78 |
| *M. conceptionense* | >50 | 25 | 25 | 3.12 |
| *M. chitae* | >50 | 12.5 | 6.25 | >50 |
| *M. gordonae* | >50 | >50 | 3.12 | >50 |
| *M. goodie* | >50 | 25 | >50 | 0.19 |
| *M. heraklionease* | >50 | >50 | 0.19 | 50 |
| *M. kyorinense* | >50 | 3.12 | 50 | 0.78 |
| *M. masisiliense* | >50 | 12.5 | 0.19 | 0.78 |
| *M. marseiliense* | >50 | >50 | >50 | 25 |
| *M. neoaurum* | >50 | 3.12 | 0.19 | 0.78 |
| *M. paregrinum* | >50 | 6.25 | 25 | 3.12 |
| *M. phocaicum* | >50 | >50 | 25 | 6.25 |

***Mycolic acid extraction:***

The protocol was designed according to the previous article. ^5^ At first, full-grown H37Rv cells in Middlebrook 7H9 broth were collected by centrifuging and washed with fresh broth. Then cell numbers were adjusted to an OD600 value of 0.2 and treated with the predetermined MIC concentrations of 0.2X, 1X, and 10X IDD-B40and1X INH. After the desired period (7 days) of treatment, cells were collected again by centrifuging, followed by washing at least twice with distilled water. From this stage, all treatments were carried out using glassware. The cells were then treated with a mixture of methanol/toluene/concentrated H_2_SO_4_ (10:10:0.4) at 50 °C for 16-18 h. The tubes were then cooled to RT, and 2 ml hexane was added, after which the tubes were shaken vigorously, then allowed to settle. The upper layer containing mycolic acid was extracted and spotted on TLC plates in equal volumes (6 µl) along with the control mycolic acid from Mycobacterium tuberculosis (bovine strain) purchased from Sigma-Aldrich (M4537-5MG). The chromatogram was developed in petroleum ether: diethyl ether (85:15). The separated mycolates were visualized after briefly dipping the TLC plates in 5% H_2_SO_4_ and charring at 100 °C in an oven for 30 min.

***RNA Isolation and RT-PCR***

We explored the gene transcript levels of 26 genes related to mycolic-acid synthesis that were used in previous articles. ^4, 6^ At first, full-grown H37Rv cells in Middlebrook 7H9 broth were collected by centrifuging and washed with fresh broth. Then cell numbers were adjusted to an OD600 value of 0.3 and treated with the predetermined MIC concentrations of 10X IDD-B40 and10X INH. After drug treatment, RNA samples from three biological replications were extracted with an RNA protect bacteria reagent kit (Qiagen) as described by the manufacturer’s protocol. RNA samples were reverse transcribed to cDNA with a cDNA synthesis kit (Bio-Rad) as described by the manufacturer’s protocol. Primer pairs were designed for PCR confirmation of genes as described in Supplementary Table S6. Copies of 16S rRNA were used to normalize the transcript levels of their respective genes.

**Supplementary Table S6:** Sequences of primers used for qRT-PCR

| **Gene symbol** | **Gene description** | **Locus tag** | **Forward primer sequence (5' - 3')** | **Reverse primer (5' - 3')** |
| --- | --- | --- | --- | --- |
| **De novo synthesis of fatty acids** | | | | |
| fas | fatty acid synthase | Rv2524c | GATGTACCACGGCAATCT | GCAATGATATTCGGCAAGA |
| **Fatty acid elongation** | | | | |
| fabD | malonyl CoA-acyl carrier protein transacylase | Rv2243 | AAACCGAGGGAATGTTGT | CTAGATCAGCGGCTTTCG |
| acpM | meromycolate extension acyl carrier protein | Rv2244 | TGTCACTCAGGAAGAAATCATTG | ATCTCGGACGGCTCGATA |
| kasA | 3-oxoacyl-ACP synthase 1 | Rv2245 | ATCCCGAGATCGACCTTG | CGAACGAGTTGTTGACTG |
| kasB | 3-oxoacyl-ACP synthase 2 | Rv2246 | ACACTGAATCTGGTAAAC | AATCCGAACGAGTTATTG |
| fabG1 | 3-oxoacyl-ACP reductase FabG | Rv1483 | TTCGGTCGAATGATATTCATAGGT | TTGGAGGCTGCGTAGTTG |
| inhA | NADH-dependent enoyl-[ACP] reductase | Rv1484 | ATGACAGGACTGCTGGAC | TACCCGTGCGATGTGAAA |
| hadA | (3R)-hydroxyacyl-ACP dehydratase subunit HadA | Rv0635 | ACGCGTGGTATTTCGAG | CTTGTAGCCGAACACACAG |
| hadB | (3R)-hydroxyacyl-ACP dehydratase subunit HadB | Rv0636 | AGGTCGGAGACCAGCTTC | GAATCGGGTTCAAGTCACC |
| hadC | (3R)-hydroxyacyl-ACP dehydratase subunit HadC | Rv0637 | ATGGCGCTCAAGACC | GACAGCTCGGGCAAAC |
| fabH | 3-oxoacyl-[acyl-carrier-protein] synthase III | Rv0533c | GGGCTGCTCAGTGTCG | CCGGTTCGGGTGTAGAT |
| **Synthesis of fatty/mycolic acid precursors** | | | | |
| accD6 | acetyl-/propionyl-CoA carboxylase subunit beta | Rv2247 | GGGTGCACGCACATCGT | CCGAGATCTGCGGGATGTAG |
| accD5 | propionyl-CoA carboxylase subunit beta | Rv3280 | CCGCTCGGCTCATTC | GACTCTTCCCTGCGTTTG |
| accA3 | bifunctional protein acetyl-/propionyl-CoA carboxylase subunit alpha AccA | Rv3285 | TGGCTAGTCACGCCG | CGGATCACCCGCACT |
| **Meromycolic acid functionalization** | | | | |
| mmaA1 | mycolic acid methyltransferase MmaA1 | Rv0645c | CGAGCGTGACGATATGAC | GCAACCCACGTCGAGTA |
| mmaA2 | cyclopropane mycolic acid synthase CmaA | Rv0644c | CCAGGCAGCAGATGGT | AATCGTCGGAGGTTGG |
| mmaA3 | methoxy mycolic acid synthase MmaA3 | Rv0643c | CGCCAAGCTGGACCTA | GGCACGCTTCATGACC |
| mmaA4 | hydroxymycolate synthase MmaA4 | Rv0642c | GACCCGACCCGAACTTA | AGCTTGTCCAGGTTGAGG |
| cmaA1 | cyclopropane mycolic acid synthase CmaA | Rv3392c | GCGCGGTGGAAAAATACGAC | AAAGCACCGATGCTGACGA |
| cmaA2 | cyclopropane mycolic acid synthase | Rv0503c | TCAACGTCATCGGCTTG | CTGGATTCGCACCTCTTTT |
| pcaA | cyclopropane mycolic acid synthase | Rv0470c | CGCGTTCGAGCACTTC | GCACGATGGTGTGCAG |
| umaA | mycolic acid synthase UmaA | Rv0469 | ACGTCATCGGTATCACGCTC | GTCGACCTTGTCCGTGAACT |
| **Mycolic acid condensation** | | | | |
| accD4 | propionyl-CoA carboxylase subunit beta AccD | Rv3799c | ATCGTCGGCATCAACGACTC | CGGTCTGGATCGGCGAATAC |
| pks13 | polyketide synthase | Rv3800c | GGTCTCCTCGATGGTCTC | CTAAACAGCGTGCGTATG |
| fadD32 | long-chain-fatty-acid--AMP ligase FadD32 | Rv3801c | GAAAAGTGGGCGAAGG | GAAGTCAGACCACAAGATGTC |
| Rv2509 | short-chain type dehydrogenase/reductase | Rv2509 | CCCATCTCGATCCTGTGCG | CAGAAATCAAGATGCCGCCG |
| **Chaperones/Heat shock** | | | | |
| hsp | heat shock protein | Rv0251c | CCGAGATCGTCAAGGATGGC | TCTTTGTCTCCGGCGTCTTG |
| **Ribosomal RNA** | | | | |
| rrs | 16S ribosomal RNA | Rvnr01 | GTGGCGAACGGGTGAGTAAC | ARGCATCCCGTGGTCCTATC |

**Supplementary References**

1. Seo, H. *et al.* In vitro activity of alpha-viniferin isolated from the roots of Carex humilis against *Mycobacterium tuberculosis*. *Pulm. Pharmacol. Ther.* **46**, 41-47 (2017).
2. Islam, M. I. *et al.* In vitro activity of DNF-3 against drug-resistant *Mycobacterium tuberculosis*. *Int. J. Antimicrob. Agents* **54**, 69-74, doi:<https://doi.org/10.1016/j.ijantimicag.2019.02.013> (2019).
3. Odenholt-Tornqvist, I. Studies on the postantibiotic effect and the postantibiotic sub-MIC effect of meropenem. *J. Antimicrob. Chemother.* **31**, 881-892 (1993).
4. Kim, S. *et al.* In vitro activity of collinin isolated from the leaves of Zanthoxylum schinifolium against multidrug-and extensively drug-resistant *Mycobacterium tuberculosis*. *Phytomedicine* **46**, 104-110 (2018).
5. Jyoti, M. A. *et al.* In vitro effect of ursolic acid on the inhibition of *Mycobacterium tuberculosis* and its cell wall mycolic acid. *Pulm. Pharmacol. Ther.* **33**, 17-24 (2015).
6. Pawełczyk, J. & Kremer, L. The molecular genetics of mycolic acid biosynthesis. *Microbiol. Spectr.* **2**, MGM2-0003-2013, doi:10.1128/microbiolspec.mgm2-0003-2013 (2014).
